# Attentional Modulation of Auditory Steady-State Responses

**DOI:** 10.1371/journal.pone.0110902

**Published:** 2014-10-21

**Authors:** Yatin Mahajan, Chris Davis, Jeesun Kim

**Affiliations:** The MARCS Institute, University of Western Sydney, Penrith, New South Wales, Australia; University of Salamanca- Institute for Neuroscience of Castille and Leon and Medical School, Spain

## Abstract

Auditory selective attention enables task-relevant auditory events to be enhanced and irrelevant ones suppressed. In the present study we used a frequency tagging paradigm to investigate the effects of attention on auditory steady state responses (ASSR). The ASSR was elicited by simultaneously presenting two different streams of white noise, amplitude modulated at either 16 and 23.5 Hz or 32.5 and 40 Hz. The two different frequencies were presented to each ear and participants were instructed to selectively attend to one ear or the other (confirmed by behavioral evidence). The results revealed that modulation of ASSR by selective attention depended on the modulation frequencies used and whether the activation was contralateral or ipsilateral. Attention enhanced the ASSR for contralateral activation from either ear for 16 Hz and suppressed the ASSR for ipsilateral activation for 16 Hz and 23.5 Hz. For modulation frequencies of 32.5 or 40 Hz attention did not affect the ASSR. We propose that the pattern of enhancement and inhibition may be due to binaural suppressive effects on ipsilateral stimulation and the dominance of contralateral hemisphere during dichotic listening. In addition to the influence of cortical processing asymmetries, these results may also reflect a bias towards inhibitory ipsilateral and excitatory contralateral activation present at the level of inferior colliculus. That the effect of attention was clearest for the lower modulation frequencies suggests that such effects are likely mediated by cortical brain structures or by those in close proximity to cortex.

## Introduction

A listener in a typical everyday situation receives multiple auditory inputs some of which may be relevant and others not. As such, the listener has to selectively attend to particular inputs and sustain this attention over time. It is through sustained selective attention that a listener is able to enhance task-relevant processing and suppress irrelevant processing [1], [2].

The neurophysiological mechanisms underlying selective auditory attention and its neural correlates have been studied using various electrophysiological methods (EEG, MEG and electrocorticography). Early research on the attentional modulation of cortical responses typically examined event-related potentials (ERPs) and paralleled the early behavioral work on aural discrimination by using simple transient stimuli, such as tone bursts and tone pips. Use of these stimuli provided a high degree of control over stimulus properties and presentation times. Results showed larger auditory P1, N1 and T-complex responses to attended stimuli [3], [4] and reduced activity to unattended stimuli [1], [5].

Recently, another neurophysiological measure, the auditory steady state response (ASSR) has been used to examine the effect of attention on neurophysiological responses underlying selective auditory attention. The ASSR consists of evoked responses from central auditory pathway and auditory cortex when presented with rapid periodic/rhythmic auditory stimuli that lead to synchronization of cortical oscillations to its frequency and phase [6]. The ASSR can be generated by modulating an auditory input (carrier signal: white noise or pure tones) either in an amplitude (AM) or frequency (FM) domain or both [7].

One benefit of using ASSR is that the modulating frequency of the auditory stream will be represented in the neural response, and multiple modulation frequencies can be used in a single stimulus paradigm to record ASSR simultaneously. Since the modulation frequencies used to record ASSR are predefined, precise frequency analyses can be performed at those frequencies. When the modulating auditory streams are attended, the attention typically influences the activity of neurons that match the temporal structure of the input and so neural responses will be tied to the timing of the attended events. These properties of ASSR enable the researchers to study the neural correlates of auditory selective attention such that the cortical responses (ASSR) are ‘tagged’ to the multiple modulation frequencies used that may be attended or unattended in a stimulus paradigm called ‘frequency tagging’.

Frequency tagging for ASSR was first introduced in a binaural interaction experiment to study the contribution of ipsilateral and contralateral pathways at the level of human auditory cortex [8]. Frequency tagging when used to evaluate auditory selective attention typically involves presentation of different auditory input in each ear with each ear modulated by a particular modulation frequency. The listener attends to sounds in one ear while ignoring the stimulus from the other ear. The ASSR is tagged for both the attended and unattended modulation frequencies from each ear. The effects of selective attention can be investigated from the resultant power of the ASSR. Furthermore, the effect of attention at different levels of auditory system can be probed with the frequency tagging paradigm by using a number of modulation frequencies. That is, the effects of attention can be assessed at different loci along central auditory pathway because such are activated by differing modulation frequencies, e.g., primary and secondary auditory cortices responsive to 4–16 Hz, the medial geniculate body of upper brainstem to 16–32 Hz and lower brainstem and other brainstem regions to 32–256 Hz AM frequency [9]–[11].

There have been only a handful of studies investigating the effect of selective attention on ASSR that have used frequency tagging. A summary of the findings and methods used in these studies is presented in Table 1. With the exception of [8], [12], all the studies shown in Table 1 employed an active attention task in which either a change in modulation frequency or carrier frequency was to be detected while paying attention to a particular ear and simultaneously ignoring the auditory stream from the other. Three general results can be drawn from these studies. First, the power of the ASSR was modulated by the deployment of attention. That is, apart from two studies [13], [14], the power of the ASSR was increased for the stream of rhythmic auditory stimuli that was attended compared to the stream that was unattended or ignored.

**Table 1:** A summary of research on attentional modulation of ASSR using the frequency tagging paradigm.

Study	Number of participants/Carrier Signal	Modulation frequencies	Effect of Attention on ASSR	Hemispheric lateralization	Task (Listening)
Linden et al., 1987	10 (5 females)/500 Hz	37 and 41 Hz	No effect	NR	Active (Change in carrier) frequency)
Fujiki et al., 2002	12 (5 females)/1000 Hz	26.1 and 20.1 Hz	NR	Left hemisphere laterality; suppression of ipsilateral responses in right hemisphere	Active (No task)
Kaneko et al., 2003	10 (4 females)/1000 Hz	39.1 and 41.1 Hz	NR	Binaural suppression of ipsilateral responses; contralateral hemisphere dominance	Active (No task)
Bidet-Caulet et al., 2007	12 (8 females)/659–784 Hz	21 and 29 Hz	Increased responses when attending; decreased when unattended	Left hemisphere laterality	Active (Target detection and localization)
Müller et al., 2009	15 (6 females)/500 Hz	20 and 45 Hz	Contralateral responses enhanced and ipsilateral responses suppressed only for 20 Hz	Left hemisphere laterality	Active (Target detection, change in modulation frequency)
Xiang et al., 2010	28 (15 females)/250–500 Hz	4 and 7 Hz	Responses enhanced by attention for each frequency tested	NR	Active (Deviant tone detection)
Lazzouni et al., 2010	15 (8 females)/1000 Hz	39 and 41 Hz	No effect of attention on ASSR power but increased ASSR amplitude (time-domain)	Right Hemisphere laterality; Binaural suppression of ipsilateral responses	Active (Target detection, change in carrier)
Bhardwaj et al., 2014	10 (2 females)/Vowels	35 and 45 Hz	Increased responses for attended frequencies	Larger responses in contralateral hemisphere	Active (Target detection)
Current Study	23 (10 females)/White noise	16, 23.5, 32.5 and 40 Hz	Contralateral responses enhanced for 16 and 23.5; ipsilateral responses suppressed for 16 Hz	No hemispheric laterality	Active (Target detection, change in modulation frequency)

Note: NR = Not Relevant.

Second, it appears that the enhancement of ASSR by attention is clearest in the hemisphere contralateral to the stimulated ear. Müller et al. [15] reported that attention enhanced the power of contralateral ASSR tagged to 20 Hz and suppressed ipsilateral 20 Hz ASSR. Bharadwaj et al. [16] found significantly increased ASSR in the hemisphere contralateral to attended sound source and only a trend of enhancement in the ipsilateral one. Ross et al. [17] also reported increased 40 Hz ASSR amplitude on attention in the contralateral hemisphere during monaural stimulation. The other studies did not report results as a function of contralateral and ipsilateral activations per se, but rather in terms of attention effects in the left or right hemisphere. Of these studies all but one [14] reported an effect of attention in left hemisphere; Lazzouni et al. [14] reported modulation of ASSR in the right hemisphere. A reason why attention might have a more potent effect contralaterally than ipsilaterally is there are more neurons and connections from subcortical structures to contralateral cortical ones [18]. It should be noted, however, that although the suppression of ipsilateral responses compared to contralateral responses is reported in a number of frequency tagging paradigms [8], [12], [14], these studies either had no attention related task or did not alternate the modulation frequencies across the ears. Alternating the frequencies and attentional load across the ears allows for the ipsilateral and contralateral auditory pathways to potentially contribute equally for each frequency providing a fairer assessment of hemispheric dominance related to attention. Indeed, in this regard, only one study [15] has employed a suitable design to properly assess the effect of attention on ASSR as a function of ipsilateral and contralateral responses in a frequency tagging paradigm.

Third, the Table 1 suggests that the modulation of ASSR by attention may depend on the modulation frequencies used; however the results are inconsistent. Müller et al. found significant attentional modulation only for 20 Hz and not for 45 Hz ASSR. Other studies have found significant attentional modulation of ASSR when tagged to 35–45 Hz modulation frequencies [14], [16]. Moreover, using an active listening oddball paradigm with 20 Hz and 40 Hz AM frequencies, Skosnik et al. [19] found increased ASSR amplitude for attended 40 Hz targets compared to 20 Hz ones. Although the existing data suggests that attentional modulation of ASSR might depend on the modulation frequencies being used in the paradigm, such a conclusion may not be appropriate as it relies on cross experiment comparisons that often involve the change of many factors. In our view then, to resolve the apparent inconsistencies in the existing research an experiment is required that systematically examines multiple modulation frequencies. Furthermore, examining multiple modulation frequencies in a single experiment is worthwhile, since no previous study has ever used more than two modulation frequencies to evaluate the effect of attention on ASSR.

In sum, attention has been found to modulate the ASSR, with the strength (direction) of this activation influenced by the stimuli input, i.e., activation in the hemisphere contralateral to the attended ear produces increased amplitude and reduced amplitude in the ipsilateral hemisphere. Further, the left hemisphere is reported to be more sensitive to attentional modulation than right. The findings are inconsistent on how different modulation frequencies may interact with the attention effect.

The purpose of the present research was to build on the above research that has used a frequency tagging paradigm with the aim of determining how selective sustained attention modulates cortical responses (ASSR) as a function of contralateral/ipsilateral activations across different modulation frequencies. The results will contribute to the growing body of literature evaluating the neural correlates of selective auditory attention by frequency tagging.

## Materials and Methods

### Ethics Statement

The methods of the present research were approved by the human research ethics committee at the University of Western Sydney. Written informed consent was obtained from each participant before the experiment.

### Participants

Twenty three participants (10 females), aged 22–35 years were recruited using notice board advertisements around the university campus. All the participants were right handed as assessed by Edinburgh handedness inventory and reported no significant neurological and psychological history. All the participants had normal hearing bilaterally with hearing thresholds of ≤15 dB HL at 500 Hz, 1000 Hz and 2000 Hz determined by screening audiometry.

### Experimental stimuli

The experimental stimuli consisted of four ‘standard’ 30 second long white noise stimuli amplitude modulated at 16 Hz, 23.5 Hz, 32.5 Hz and 40 Hz with 100% modulation depth sampled at the rate of 44,100 Hz. The stimuli were ramped with 20 ms rise time at the onset and 20 ms fall time at the offset to avoid clicks. The white noise was used as a carrier signal in order to evoke reliable and robust ASSR. The broadband signal when used to elicit ASSR, produces larger magnitude of ASSR as compared to pure tone and band-limited noise as carrier signals [20]. The stimuli were created using the signal processing toolbox of MATLAB and were presented at a comfortable level around 70–75 dB SPL for all the participants. The experiment was completed in two sessions. In one session, the participants were presented with 16 Hz and 23.5 Hz AM stimuli and in the other session the 32.5 Hz and 40 Hz AM stimuli. The two sessions of recording were counterbalanced between the participants.

During each session the participants were presented each of the two stimuli in each of their two ears dichotically such that the one ear was stimulated by 16 Hz or 32.5 Hz and other ear by 23.5 Hz or 40 Hz modulated white noise. The ear of stimulation was counterbalanced equally between the two sets of stimuli such that a particular amplitude modulated stimulus was presented equally to both left and right ears and at the same time the opposite ear was stimulated with a different stimulus.

The experiment also consisted of ‘target’ stimuli, in which the modulation frequency changed multiple times during 30 seconds of stimulation (based on paradigm used by Müller et al.). The stimuli with 16 Hz and 23.5 Hz amplitude modulation changed to 40 Hz modulation for 2 seconds, either two, three or four times within 30 seconds of stimulation for target stimuli before returning to original modulation rate. The target stimuli with 32.5 Hz and 40 Hz amplitude modulation changed to 12.5 Hz modulation frequency. The target stimuli were also counterbalanced equally between ears in two sessions of recording. The two frequencies using as stimulus pairs were selected so that there was a relatively small difference between them; this was done to minimize involuntary switching of attention to one of the frequencies. That is, as part of a pilot study conducted to determine the best combination of modulation frequencies, we found that when the difference in frequencies was large (e.g., 16 and 40 Hz) then participants' attention involuntarily switched to higher modulation frequency (i.e. the 40 Hz). Further, even if the stimulus intensity was equalized across all the modulation frequencies, the perceived loudness of the higher modulation rate stimulus was greater than the lower one and this plausibly would induce an involuntary attention switch towards the high modulation frequency. To minimize this possible effect, we selected modulation frequencies pairs that were closer together (i.e., 16 and 23.5 Hz, 32.5 and 40 Hz) and this combination did not produce any involuntary attention switches.

### Experimental procedure

**Figure 1:**
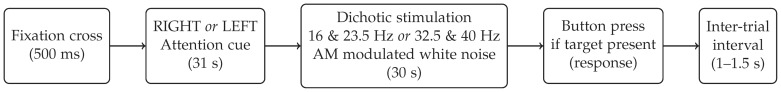
A depiction of the trial sequence.

An attention switch paradigm was used to direct attention to the stimulus presented in the designated ear for the dichotic stimuli, (see Fig. 1). The trial started with a fixation cross presented at the center of the screen for 500 ms. Then, a cue in the form of the words ‘RIGHT’ or ‘LEFT’ appeared on the screen indicating the participants which ear to attend to and after 1 second the set of paired stimuli (16 or 23.5 Hz; 32.5 or 40 Hz) were presented for 30 seconds. The cue remained on the screen for the duration of the stimuli. The participants were instructed to attend to the ear cued and press the response button as fast as they can whenever they heard a change in modulation frequency of the stimuli (‘target’). A total of 72 trials were presented in one experimental session. Out of these 72 trials, 48 were standard trials that contained no targets and 24 trials had targets in them. In 12 target trials, the location of the targets and attention cue were same, i.e., if the cue was ‘RIGHT’, the target also appeared during right ear stimulation; these trials represent the ‘congruent’ condition. The ‘incongruent’ condition consisted of the remaining 12 target trials in which the cue and the stimulation ear were at opposite locations. The reaction times for detecting the targets were recorded and slower reaction times were expected in the incongruent condition compared to the congruent one (assuming that attention manipulation had been successful).

### Electrophysiological recording

The participants were seated on a comfortable chair while the electrode cap was fitted. Prior to the fitting of the electrode cap, the scalp of each participant was combed to reduce the time taken to achieve the optimal scalp electrode impedance [21]. The raw electroencephalograph (EEG) was recorded with a BioSemi Active-Two amplifier system (BioSemi, Amsterdam, Netherlands). The 64 Ag-AgCl electrodes were mounted on a nylon electrode cap according to the international standard 10-10 system [22]. There were two electrodes on the electrode cap (CMS & DRL) which served as online references. Six additional electrodes were also placed on the participants. Four of them were bipolar electrodes placed above and below the left eye and outer canthi of both the eyes to monitor vertical and horizontal eye movements (EOG channels) respectively and two electrodes were placed on two mastoids. The raw EEG recording was sampled at 512 Hz with online band-pass filtering of.05–200 Hz. This raw EEG data was stored for every participant for later offline analysis.

### EEG data analysis

The pre-processing and analysis of the stored raw EEG data was carried out using EEGLAB version 10 [23] and custom written functions in MATLAB (The Mathworks, Natick, MA). Initially, any obvious artifact was removed after visually inspecting the data. Then the EEG data was re-referenced to the average of both the mastoids. The resultant EEG activity was band-pass filtered (1 Hz high pass and 70 Hz low pass; 12 dB per octave roll-off). The filtered data then was epoched into a pre-stimulus period of 200 ms and post stimulus period of 30 seconds. Only the standard trials were included in the EEG analysis. The epoched data then was subjected to *runica*, an ICA (Independent component analysis) algorithm incorporated in EEGLAB to detect and remove eye blinks, horizontal eye movements and other artifacts (muscle, line noise artifacts). The ICA algorithm resulted in 64 components and based on the scalp topography, activity power spectrum and activity over trials, the artifactual components were identified and removed from the EEG data. To remove the effect of onset and offset ERP responses, the epochs were averaged from 1 s to 29.5 s in the ICA corrected epochs to form ASSR responses. The averaged ASSR responses were calculated for each modulation frequency in four conditions namely, ‘right attended’, ‘right unattended’, ‘left attended’ and ‘left unattended’.

These time domain ASSR responses were then subjected to a Fourier transformation using a custom written MATLAB script to convert them into frequency domain (FFTs). The FFTs were used to calculate the absolute ASSR power for each condition for every modulation frequency at the two most lateral electrodes (T7 and T8) on the scalp depicting left and right hemispheric activity respectively. These two electrodes were selected as the greater distance between the locations of T7 and T8 makes them the best electrodes to compare neural activity between the two hemispheres, as opposed to using two fronto-central electrodes. To determine if the ASSR at the modulation frequencies of interest evoked at T7 and T8 was significant across participants, the mean absolute power at the two neighboring frequencies on the either side of the modulation frequency were computed and compared with the power at the modulation frequency of interest using a t-test in the resultant FFTs. Results revealed that across all the conditions the power of the ASSR at the frequency of interest was a significant response.

### Data analysis

To determine how attention modulates the ASSR as a function of modulation frequency and hemisphere by ear of stimulation (ipsilateral and contralateral), the data on the absolute power of the ASSR obtained through FFT was subjected to a 4 (‘modulation frequency’; 16 Hz, 23.5 Hz, 32.5 Hz & 40 Hz)×2 (‘stimulation ear’; left vs. right)×2 (‘attention’; attended vs. unattended)×2 (‘hemisphere by stimulation’ (ipsilateral vs. contralateral)) within participant factorial ANOVA. The reaction times obtained in the target detection task were also analyzed with a 4×2×2 repeated measures ANOVA, with ‘Frequency’ (four modulation frequencies), ‘congruency’ (congruent vs. incongruent) and ‘stimulation ear’ (left vs. right) as within participant factors. This analysis provided an index of the differential attention paid to the experimental stimuli. For both above mentioned ANOVA analyses, wherever the assumption of sphericity was violated the Greenhouse-Geisser correction was applied. The significant results obtained are reported in the section below.

## Results

### Reaction times

Table 2 shows the reaction times obtained for four modulation frequencies across congruent and incongruent conditions. The ANOVA results indicated that congruency significantly altered the reaction times (*F*(1,17) = 24.77, *p<.001, η_p_^2^* = .56) and the modulation frequencies and the ear of stimulation had no significant main effect on reaction times. As evident from Table 2, reaction times were slower when participants attended to targets in the incongruent conditions across the different modulation frequencies. Similar reaction times between right and left ears and slower response times to targets in incongruent conditions across modulation frequencies when considered together, suggest that participants paid and sustained attention to the appropriate ear as instructed at the beginning of each trial.

**Table 2:** Mean reaction times for targets across modulation frequencies for the congruent and incongruent condition. Standard deviations are given in parentheses.

Frequency	Congruent		Incongruent	
	Left	Right	Left	Right
16 Hz	493 (134.27)	489 (116.85)	531 (138.71)	574 (168.75)
23.5 Hz	492 (122.04)	535 (144.49)	559 (139.36)	584 (152.18)
32.5 Hz	532 (152.54)	532 (133.57)	572 (127.33)	596 (117.65)
40 Hz	510 (153.30)	501 (149.97)	587 (141.44)	545 (142.54)

### ASSR

**Figure 2:**
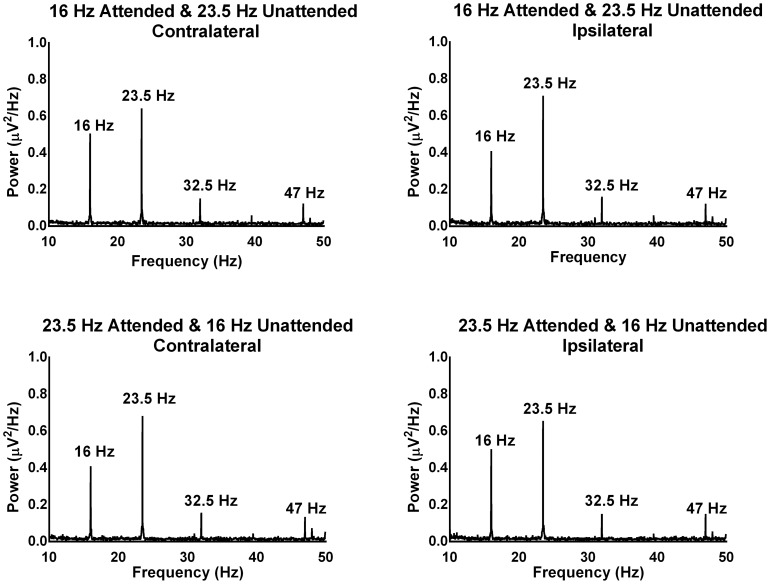
The grand mean FFTs for 16 and 23.5 Hz modulation frequencies during attended and unattended conditions across ipsilateral and contralateral activations combined from the two electrodes (T7 & T8). The power in the FFTs is an absolute value expressed in terms of squared microvolts per every 1 Hz of frequency (µV^2^/Hz). The first harmonics for both 16 and 23.5 Hz are also shown.

**Figure 3:**
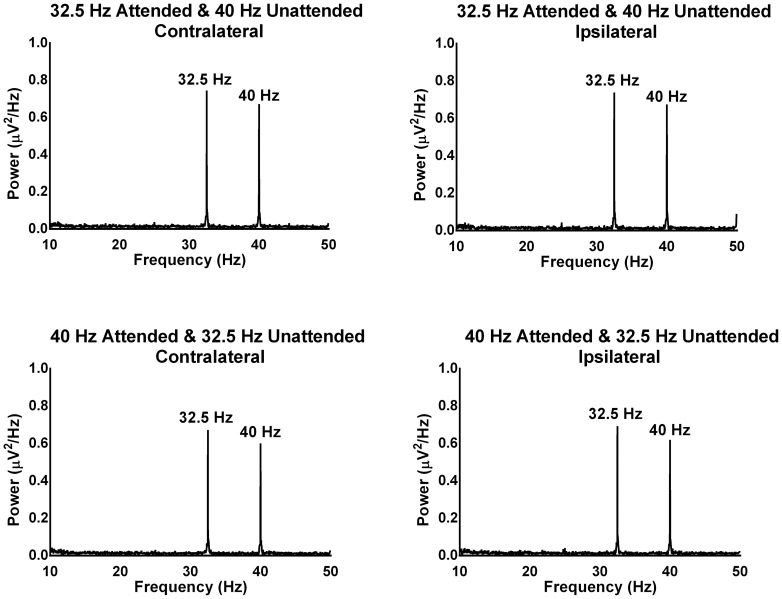
The grand mean FFTs for 32.5 and 40 Hz modulation frequencies during attended and unattended conditions across ipsilateral and contralateral activations combined from the two electrodes (T7 & T8). The power in the FFTs is an absolute value expressed in terms of squared microvolts per every 1 Hz of frequency (µV^2^/Hz).

Fig. 2 and Fig. 3 show the grand mean FFTs of the ipsilateral and contralateral activation patterns for the 16 Hz/23.5 Hz and 32.5 Hz/40 Hz modulation frequencies respectively as a function of attention (i.e., presented to the attended and unattended ear). The presence of clear and robust peaks in the FFTs indicates that the multiple frequencies presented had the desired effect of driving auditory responses at those frequencies. The results of repeated measures ANOVA revealed that there was a significant three-way interaction between frequency, attention and activation pattern, *F*(3,66) = 5.76, *p* = .005, η*_p_^2^* = .20 (there was no significant main effect of modulation frequency, attention, ear of stimulation or hemisphere by stimulation). To understand this complex three way interaction, first the ipsilateral and contralateral activations were combined from the two ears for each attended and unattended condition across the four modulation frequencies. Then, a follow up two-way ANOVA was computed with factors ‘attention’ (attended & unattended) and ‘hemisphere by stimulation’ (ipsilateral & contralateral) for each modulation frequency (16 Hz, 23.5 Hz, 32.5 Hz & 40 Hz).

**Figure 4:**
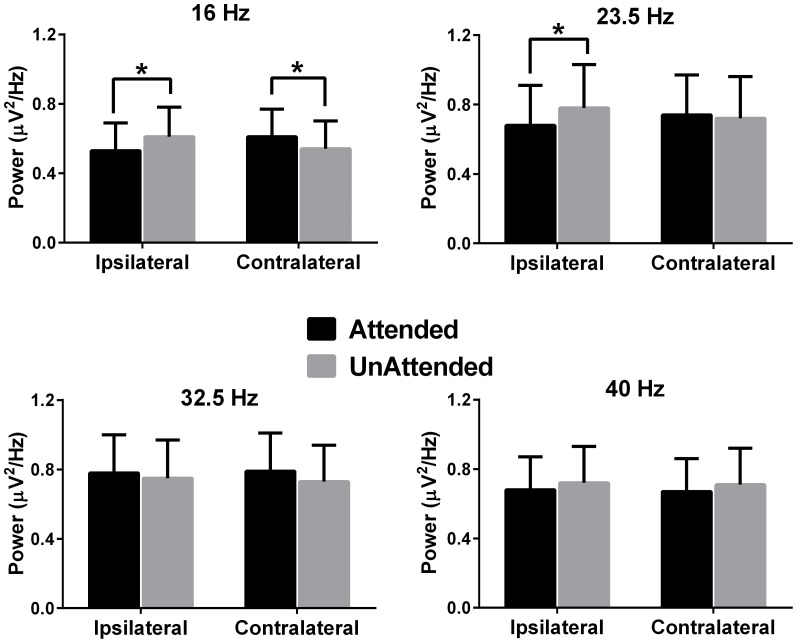
The bar graphs represent the absolute power measured from FFTs, when white noise was modulated with a particular modulation frequency and was attended or unattended. The bars represent neural activity from the ipsilateral and contralateral activations combined from T7 and T8 sites. An asterisk above the bar graphs indicates significant differences at *p*<.05.

The results of this ANOVA revealed that there was no significant main effect of attention and hemisphere by stimulation across all the modulation frequencies. There was a significant interaction between these factors for the 16 Hz (*F*(1,22) = 14.40, *p* = .001, η*_p_^2^* = .39) and 23.5 Hz (*F*(1,22) = 10.47, *p* = .004, η*_p_^2^* = .32) modulation frequencies. To investigate this interaction, subsequent one-way ANOVAs were conducted on each 16 Hz and 23.5 Hz modulation frequency. For the 16 Hz modulation, the power of the ASSR for attended compared to the unattended stimuli was significantly suppressed for ipsilateral activation (*F*(1,22) = 9.17, *p* = .005) and significantly enhanced for contralateral activation (*F*(1,22) = 9.93, *p* = .006). A similar significant suppression on attended ipsilateral stimulation was found for 23.5 Hz, (*F*(1,22) = 8.46, *p* = .008), but attention did not affect contralateral stimulation (*F*(1,22) = .21, *p* = .64). For 32.5 Hz and 40 Hz AM frequencies, attention did not alter the power of the ASSR, although the change in power was in the enhancement direction for 32.5 Hz and suppression for 40 Hz for both ipsilateral and contralateral stimulation, respectively. These results are illustrated in Fig. 4. The complete statistical analysis has been summarized in supplementary Table S1.

## Discussion

The aim of the present research was to determine the extent to which auditory sustained selective attention modulates the strength of ASSR for ipsilateral and contralateral stimulation across different modulation frequencies. The results from the behavioral task demonstrated an effect of selective attention: responses were slower to incongruent (target presented on the unattended side) versus congruent targets depicting successful manipulation of attention. Below we consider the effects of this allocation of attention on the ASSR as a function of the various experimental manipulations.

### Attentional modulation of ASSR

To our knowledge, this is the first study that has used a frequency tagging paradigm with more than two modulation frequencies to test the effect of attention on ASSR. This design allows for the direct comparison of the effect of attention on ASSR by multiple modulation frequencies. Our results indicated that attention significantly modulated the power of ASSR and, except for two studies [13], [14] listed in Table 1, our results are in general agreement with other studies. The inconsistencies with the results of the studies in Table 1 may be explained by the differences in the experimental tasks used to manipulate the attention. The participants in the current study (and in [15], [24]) attended to a change in the modulation frequency or the temporal envelope of stimuli which revealed modulation of ASSR. In the two studies that failed to show any significant modulation of ASSR power [13], [14], the participants' task was to attend to changes in carrier frequency (or to spectral change). This difference in task raises the possibility that the attentional modulation of the ASSR requires that attention be directed to modulation frequency change rather than a change in the carrier frequency. In other words, it appears that attending to the stimulus parameter that drive the ASSR (modulation frequency) is essential for its modulation whereas attending to any other stimulus change (carrier frequency) is not. Further, we found that the attentional modulation of ASSR depended on the modulation frequencies and pattern of activation, which are discussed below.

### Attentional modulation of ASSR across modulation frequencies and hemispheric dominance

The results indicated that selective attention influenced the power of ASSR for the 16 and 23.5 Hz modulation frequencies whereas this was not the case for the two other modulation frequencies used. These results are in general agreement with two studies (see Table 1), that also reported significant modulation by attention for 20–29 Hz ASSR [15], [24]. It has been reported that white noise when amplitude modulated using a range of modulation frequencies (4–256 Hz) tends to preferentially stimulate different parts of central auditory pathway [9]. That is, the primary auditory cortex and the neural structures closer to the cortex like the medial geniculate body and a portion of lower brainstem appear to be more sensitive to 16–32 Hz AM frequencies, whereas the lower brainstem structures are more responsive to >40 Hz AM frequencies. Based on this selective responsiveness of neural structures within the auditory system, we suggest that assemblies more proximal to the cortex are likely to be more susceptible to attentional effects than those more distal from the cortex. Such a differential sensitivity would result in lower modulation frequencies being the ones most likely to show an attention effect. This claim that the activity of neural structures higher in auditory system will be more open to attention modulation than those located down the auditory hierarchy is supported by a series of studies that have reported no effects of attention on auditory brainstem responses [1].

The results of the present study are inconsistent with the results of a number of previous studies that have found significant modulation of ASSR by attention for modulation rates between 35–45 Hz [16], [17], [19]. In line with the explanation proposed by Müller et al to clarify the lack of attentional modulation of 45 Hz ASSR in their study, this discrepancy might have stemmed from methodological differences between the studies. For example, Skosnik et al. found attention effects only for 45 Hz ASSR and not 20 Hz ASSR (they also used binaural stimulation of these frequencies with clicks as carrier signals in an oddball paradigm). Ross et al. used only monaural stimulation in their target detection task for 40 Hz ASSR (change in modulation frequency) and used a concurrent visual control task for unattended condition, thus involving two modalities in contrast to the present study that investigated effect of attention with in a single modality. Lastly, in a MEG study, Bharadwaj et al used rapid presentation of vowels (A, E, I, O and U) at 35 and 45 Hz spatially to the participants two ears based on his/her head related transfer functions. The task was to attend to one spatial stream and count a particular vowel in that stream and like Ross et al a visual control task was used as an unattended condition.

In the current study, there was no separate unattended control condition as we considered the modulation frequency presented opposite to the attended ear to be unattended. Furthermore, we suggest that in examining the effects of auditory selective attention on ASSR, it is important that the unattended or control condition should only involve the auditory modality. That is, a visual attention control task in an auditory selective paradigm may stimulate additional cortical areas and pathways apart from auditory ones; hence the results will not be specific to auditory stimulation alone. The differences in the stimulation pattern (binaural vs. monaural vs. dichotic), unattended conditions (visual control vs. attention switch) between previous studies [16], [17], [19] and the present study might indicate that the current results are specific to the experimental manipulations we used and as such do not conflict with previous results per se. In support of the idea that modulation effects might be influenced by the precise paradigm used, our paradigm was based on Müller et al's paradigm in terms of having an attention switching task between the ears and to detect a change in modulation frequencies. They also reported attention effects only for 20 Hz and not 45 Hz modulation frequencies.

As shown in Table 1, previous research found significant attentional modulation of ASSR either in left hemisphere and right ear [15], [17], [24] or in the right hemisphere [14]. Since the task in the current study was to detect a change in the modulation frequency, i.e., a change in the temporal envelope, left hemisphere laterality was expected for the attentional modulation as opposed to right hemisphere when a change in carrier frequency was to be detected (spectral change; [14]). However, the results revealed neither an ear effect nor any hemispheric laterality effect. The reason for the lack of an influence of hemispheric laterality is not clear. A notable difference between our study and previous ones is that we used white noise as a carrier signal, whereas previous studies used pure tones of different frequencies (see Table 1). We employed white noise as a carrier signal to evoke reliable, robust and larger ASSR responses since the white noise activates a larger region of the basilar membrane, which in turn provides more sensory input to higher cortical structures [25]. Except for one study [16] that used vowels as stimuli to evoke ASSR, other studies used pure tones as carrier signals and have reported attentional modulation of ASSR in either left or right hemisphere. The type of carrier signal (broadband or pure tone) or the band width of the carrier signal (large for white noise and small for pure tone) might determine the laterality of the ASSR when modulated by the attention. In line with the findings of previous studies that have showed hemispheric laterality of ASSR, it is plausible that that narrow band carrier signal when used to evoke ASSR is more likely to elicit clear hemisphere laterality during attentional modulation as compared to a broadband signal as in present study. Support to this proposal comes from a recent experiment [26] on hemispheric laterality of various auditory processing tasks such as gap detection, frequency discrimination and intensity discrimination. The results showed that no hemispheric lateralization was found for psychoacoustic thresholds obtained from these tasks with broad band stimuli whereas clear left hemisphere laterality was found for pure tones.

Additionally, it has been reported that in conventional central masking scenarios, the power of the ASSR reduces in both the hemispheres when evoked monaurally in the presence of contralaterally presented continuous white noise [27]. Applying similar principles to the results of the present study, it is possible that the continuous white noise stimulation from each ear might have reduced the power of ASSR in both hemispheres and in effect eliminated any hemispheric differences that might have been evident during attentional modulation. Though, it must be noted that this account is speculative and further systematic investigations would be needed to confirm this.

### Attentional modulation of ASSR across ipsilateral and contralateral activations

It was found that attention differentially affected ASSR power as a function of ipsilateral versus contralateral stimulation. When attended, the ASSR was suppressed for ipsilateral stimulation at modulation frequencies of 16 and 23.5 Hz and enhanced for contralateral stimulation at 16 Hz (the effect for 23.5 Hz was in the enhancement direction but was not significant). In general, these results agree with those of Müller et al who also reported similar suppression and enhancement effects for 20 Hz frequency for ipsilateral and contralateral stimulation, respectively and did not find any significant effects for the 45 Hz ASSR.

Müller et al. explained these findings based on an experiment by Staines et al. [28] that examined the somatosensory system and where an enhancement for contralateral and suppression for ipsilateral stimulation was found. In the study by Staines et al it was found that task relevant stimulation increased the BOLD response in the somatosensory cortex contralateral to stimulation and decreased it in the ipsilateral one. Staines et al. pointed out that this was unexpected since the suppression in the ipsilateral cortex occurred in response to task-relevant somatosensory stimulation (the task was to detect a change in frequency of vibrotactile stimulation, no matter on what side it occurred). The explanation that was given for this inhibitory effect was that, making the ipsilateral cortex less responsive would mitigate the effects that potentially conflicting inputs would have on a behaviorally relevant task. Müller et al. explained their ASSR modulation assuming that information is likely to be more relevant when processed by the contralateral mechanisms than when processed by ipsilateral ones. That is, relevant contralateral activity gets enhanced by attention whereas ipsilateral activity gets suppressed. A problem with this account is that the basis of the assumption that relevant and irrelevant stimulation map to contralateral and ipsilateral mechanisms has not been made clear. Also, the ipsilateral effect has not been found across stimulation frequencies. For example, Bhardwaj et al. found an enhancement effect for contralateral ASSR for 35 Hz and 45 Hz modulations but no ipsilateral effect for these modulation frequencies.

There are several other approaches for explaining the finding that attention leads to contralateral enhancement and ipsilateral suppression. One approach builds on two proposals that have been made about findings using dichotic listening. First, it has been reported that the dichotic listening leads to a cortical level competition between the two auditory inputs from the two ears [8], [12]. This competition may lead to either summation or suppression of neural responses. Fujiki et al. [8] and Kaneko et al. [12] found suppression of ipsilateral activity in both the hemispheres during dichotic listening when compared with monaural stimulation. While there was no task associated with Fujiki et al's and Kaneko et al's frequency tagging paradigms, the listeners were attending to the stimuli. The present study which employed a task-relevant dichotic listening frequency tagging paradigm would have produced similar ipsilateral suppressive effects on ASSR power. The binaural rivalry from the two ears in response to dichotic stimulation led to the suppression of ipsilateral activity during attention. Secondly, it is well-known that during dichotic listening, there is a shift of hemispheric balance towards the hemisphere contralateral to the stimulation owing to the relatively large number of contralateral neural connections [29]. This property might have led to enhanced and larger ASSR on attention for the contralateral side during dichotic stimulation in the present study. It should be pointed out that the suppression and summation of ASSR reported for dichotic listening in Fujiki et a. [8] and Kaneko et al. [12] was with higher modulation frequencies (26, 39 & 41 Hz) than those used in present study. Given this, and the lack of ipsilateral suppression by Bhardawaj et al. [16] and that the differential effects of attention in the present study were present only for two of the four tested modulation frequencies, it would appear that attentional effects on the ASSR are complex and likely depend on the experimental procedures used to manipulate attention. We therefore suggest that our interpretation of attentional effects on ASSR based on dichotic listening should be viewed with some caution.

A slightly different explanation suggests that the pattern of enhancement and inhibition as a function of neural connectivity may be ultimately due to the anatomical arrangement and function of the inferior colliculus (IC). The IC contains EI cells (excitatory-inhibitory cells) that enhance contralateral input and suppresses the ipsilateral input [30], [31]. This arrangement may influence processing at cortical regions and result in relative inhibition of ipsilateral activity and enhancement of contralateral activity. It is known that, selective attention enhances the underlying neuronal output and increases the synchronization of the local neuronal output [32]. Accordingly, during selective attention the overall neural firing of the EI cells at the level of ICs will also increase; with EI cells being excitatory and inhibitory in nature, the increased output will be inhibited at ipsilateral cortex and enhanced on the contralateral cortex. Indeed, bilateral activation at the level of ICs in an auditory selective attention task, where the participants had to selectively attend to an increasing or decreasing pitch in one ear and ignore the stimulus in the other, has been reported [33]. Rinne et al. [33] observed increased activations at the level of ICs, while attending the stimuli contralaterally than ipsilaterally. These findings suggests that on attention, the selective auditory processing at cortical level is mediated both by the increased neuronal output at the level of ICs and by the excitatory-inhibitory properties of the EI cells which alter the contralateral and ipsilateral activations at cortical level.

## Conclusions

The results of the present study indicate that modulation of ASSR by selective attention: 1) depends upon the modulation frequencies used in the paradigm (with 16 and 23.5 Hz being modulated by attention). 2) Also depends upon the pattern of activation at the cortical level, with contralateral activations from either ear enhanced the ASSR and ipsilateral activations suppressed the ASSR. 3) Can be probed efficiently using a frequency tagging paradigm in which participants monitor a change in the temporal envelope.

The results of the present study contribute to the limited but emerging body of research on auditory selective attention using an ecologically valid frequency tagging scenario and also stress the importance of replication of research. Future studies may usefully look into the attentional modulation of low frequencies (3–8 Hz) to which primary and secondary auditory cortices are highly responsive. Based on current findings we would predict a significant modulation of low frequency ASSR as well. Also an experimental comparison between different carrier stimuli is required to examine the relationship between ipsilateral/contralateral activations and attention.

## Supporting Information

Table S1 **The ANOVA results.** The significant interactions are presented in bold.
(DOCX)
