# Supplementary material for: Attentional Modulation of Auditory Steady-State Responses
Source: PLoS One. 2014 Oct 21;9(10):e110902. doi: 10.1371/journal.pone.0110902 (PMC4205007; doi:10.1371/journal.pone.0110902)
Supplement: Table S1 — The ANOVA results. The significant interactions are presented in bold. (DOCX) [file pone.0110902.s001.docx]

Table S1. The ANOVA results. The significant interactions are presented in bold.

| **Variables** | **F values** | **p** | **ŋ_p_^2^** |
| --- | --- | --- | --- |
| Modulation Frequency | F(3,66) = 0.77 | 0.51 | 0.03 |
| Attention | F(1,22) = 0.69 | 0.41 | 0.03 |
| Stimulation ear | F(1,22) = 0.16 | 0.68 | 0.007 |
| Hemisphere by Stimulation | F(1,22) = 0.11 | 0.73 | 0.005 |
| Modulation Frequency x Attention | F(3,66) = 1.91 | 0.13 | 0.08 |
| Modulation Frequency x Hemisphere by Stimulation | F(3,66) = 1.02 | 0.38 | 0.04 |
| **Hemisphere by Stimulation x Attention** | **F(1,22) = 12.52** | **.002** | **0.36** |
| **Modulation Frequency x Hemisphere by Stimulation x Attention** | **F(3,66) = 5.76** | **.001** | **0.20** |
| Modulation Frequency x Stimulation Ear | F(3,66) = 0.95 | 0.41 | 0.04 |
| Hemisphere by Stimulation x Stimulation Ear | F(1,22) = .37 | 0.54 | 0.01 |
| Modulation Frequency x Hemisphere by Stimulation x Stimulation Ear | F(1,22) = 0.37 | 0.77 | 0.01 |
| Attention x Stimulation Ear | F(1,22) = 0.00 | 0.99 | 0.00 |
| Modulation Frequency x Attention x Stimulation Ear | F(3,66) = 2.33 | 0.08 | 0.09 |
| Hemisphere by Stimulation x Attention x Stimulation Ear | F(1,22) = 0.54 | 0.47 | 0.02 |
| Modulation Frequency x Hemisphere by Stimulation x Attention x Stimulation Ear | F(3,66) = 1.86 | 0.14 | 0.07 |
